# A Comparative Analysis of the Critical Resilience and Gender Equity of Mothers Impacted by Albinism: Promising Practices From Tanzania and South Africa

**DOI:** 10.1002/puh2.70239

**Published:** 2026-05-04

**Authors:** Sheryl Reimer‐Kirkham, Barbara Astle, Ikponwosa Ero, Lori Beaman, Maretha de Waal, Bonny Ibhawoh, Elvis Imafidon, Jennifer Kromberg, Ronell Leech, Nomasonto Mazibuko, Innocentia Mgijima‐Konopi, Ramadimetja Shirley Mooa, Dianah Msipa, Perpetua Senkoro, Sonya Sharma, Wisdom Tettey, Mpho Tjope, Boateng Wiafe, Meghann Buyco, Emma Strobell

**Affiliations:** ^1^ Trinity Western University Langley British Columbia Canada; ^2^ Africa Albinism Network Dar es Salaam Tanzania; ^3^ University of Ottawa Ottawa Ontario Canada; ^4^ University of Pretoria Pretoria South Africa; ^5^ McMaster University Hamilton Ontario Canada; ^6^ University of London London UK; ^7^ University of Witwatersrand Johannesburg South Africa; ^8^ Albinism Society of South Africa Johannesburg South Africa; ^9^ Tanzania Human Rights Defenders Coalition Dar es Salaam Tanzania; ^10^ University College London London UK; ^11^ Carleton University Ottawa Ontario Canada; ^12^ Albinism Advocacy for Access Delportshoop South Africa; ^13^ Operation Eyesight Ghana Accra Ghana

**Keywords:** albinism, critical ethnography, gender equity, human rights, resilience

## Abstract

**Background:**

Women bear the brunt of human rights violations faced by persons with albinism, especially in sub‐Saharan Africa. In addition to insufficient access to skin and eye care, increased rates of skin cancer, stigma and discrimination, and threats of mutilation and murder as they are reportedly trafficked for economic gain, they face heightened gender‐based violence linked to misbeliefs about albinism. This study explored through a human rights lens the resilience of mothers affected by albinism, at the intersection of gender, colorism, and religion in Tanzania and South Africa.

**Methods:**

The critical ethnography involved participant observation, interviews, and sharing circles with 97 participants, including mothers and key informants such as albinism advocates, health and social workers, community leaders, and policy makers. In Tanzania, we conducted fieldwork in Mwanza and Dar es Salaam; in South Africa, in the provinces of Northern Cape and Gauteng. We present a comparative analysis of the two countries.

**Results:**

The study shows that critical resilience comes about through social relationships, collective practices, and identities present in communities (local and national). We identified six promising practices that created conditions to strengthen gender equity: In Tanzania, peer support groups build capacity as human rights defenders; international non‐governmental organizations (NGOs) fill gaps and advocate; and faith leaders act as advocates. In South Africa, progressive health system and policy frameworks, genetic counseling and health education equip families, and traditional healers act as advocates.

**Conclusion:**

The promising practices distilled from the Tanzanian and South African cases should be considered for broader uptake, importantly with local adaptations.

## Graphical abstract

Women bear the brunt of human rights violations faced by persons with albinism. Our ethnographic study showed that critical resilience comes about through social relationships, collective practices, and identities present in communities (local and national). We identified six promising practices in Tanzania and South Africa to strengthen gender equity.

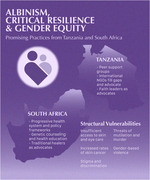

## 1 Introduction

A much‐needed shift has emerged in health and social sciences studies to incorporate strengths‐based approaches for people who experience health inequities on account of social and structural determinants of health. One site of such necessary action and the focus of this study is albinism, a noncontagious group of related conditions resulting from genetic mutations that cause a deficiency in the production of melanin, a pigment found in the hair, skin, and eyes [1, 2]. A common characteristic of persons with albinism (PWA) is the paleness of their skin and eyes, making them hypervisible in an African community [3]. The health implications of albinism include visual impairment and sensitivity to the damaging effects of the solar and ultraviolet radiation of the sun [4, 5, 6]. The prevalence of albinism varies geographically and generally is not well documented [7]. As a worldwide rate, it is reported that albinism occurs in approximately 1 in 17,000; in Africa, this prevalence rate jumps to 1 in 3900 [7]. Most albinism research has focused on genetics, with some other research addressing the everyday experience of dealing with vision and skin disorders and the psychological effects of the stigma and discrimination encountered by PWA from the moment of birth until death [8, 9, 10, 11]. In some African regions, violence (sexual violence, mutilations, and murder) and threat of violence have meant those living with albinism face a “reign of terror” [12, 13].

Women and children carry the brunt of these challenges [14, 15, 16]. Albinism adds to the intersecting structural vulnerabilities [17] that many African women navigate [18, 19]. Structural vulnerability is a social positionality that imposes physical‐emotional suffering on specific groups and individuals in a predictable, structural manner based on class, cultural, gender, and racial discrimination. Women and girls with albinism are at increased risk of experiencing gender‐based violence and sexual assault [8, 9]. Those who deliver babies with albinism are believed to be cursed or to have committed a misdeed (e.g., infidelity, conception during menstruation, touching someone with albinism while pregnant) and thus bear the blame for their child having the condition [8, 9, 20, 21]. These women often face rejection by their partners, their partners’ families, and their communities, exposing women and their children with albinism to the cumulative effects of ostracism and poverty [1, 5, 18, 22, 23]. A few studies have shown how community support, drawing on positive spiritual beliefs for causation narratives, and education can lead to resiliency, increased help‐seeking, and improved health and social outcomes [11, 24]. Little research has focused primarily on mothers impacted by albinism, despite the gender and health inequities they face, and the social transformation that can come about when they are empowered [15, 25]. To address this gap, our international research‐advocacy‐policy network (see www.motheringandalbinism.com; https://www.ohchr.org/en/documents/thematic‐reports/ahrc4062add2‐round‐table‐human‐rights‐and‐albinism‐seeking‐consensus‐and) conducted a critical ethnographic [26] study with the purpose of exploring the resilience of mothers affected by albinism, at the intersection of gender, colorism, and religion, and through a human rights lens in three African countries, Tanzania, South Africa, and Ghana. In this article, our comparative analysis focuses on Tanzania and South Africa (the COVID‐19 pandemic limited data collection in Ghana) to demonstrate how intersecting factors can weave a net of security for critical resilience, even as mothers and PWA enact courage and resist stigma, discrimination, and violence. Such promising practices are our focus here.

The last decade has seen the concept of resilience (as the process and outcome whereby someone succeeds despite adverse conditions [27]) taken up across sectors and disciplines, from psychology to ecology, and across individual and societal levels. The concept has become increasingly nonspecific as it is picked up by governments, sometimes in a neoliberal offloading of state obligations (e.g., emphasizing community and self‐reliance while reducing social services) [27, 28]. In contrast, a critical approach to resilience prompts a detailed accounting of structural constraints (particularly poverty, geography, and gender inequality) and the roles of government and non‐governmental organizations (NGOs) as determinants of resilience [27]. Moreover, critical resilience [29] draws our attention not just to everyday survival, but also to struggle, resistance, and reinvention. With its emphasis on relations between individuals, between institutions and individuals, and among institutions, critical resilience highlights the agency of individuals and collectives, even those who are highly marginalized, such as mothers impacted by albinism. To foster a critical interpretation of resilience, we relied on theories of intersectionality [30, 31], feminism [32, 33], and decolonialism [34, 35].

## 2 Methods

Tanzania, South Africa, and Ghana were chosen as settings because of their higher incidence of albinism; reports of human rights violations in these regions; and our existing research relationships. Our choices were also oriented to variation in our overall sample, as the countries have different histories, infrastructures, health and social outcomes, and approaches to albinism—all of which bring into relief what might be promising practices. Existing research relationships facilitated our identification of research focus and study design, particularly in the context of Global North:South partnering [36]. Local researchers from African universities and community organizations were involved in all aspects of ethnographic data collection, and cultural liaisons facilitated local access and contextualization. Data collection consisted of sharing circles (guided by local researchers and in keeping with Indigenous research [33], sharing circles were employed as an open‐structured, conversational style methodology that respected individual story sharing while weaving a collective narrative), in‐depth semi‐structured interviews, and participant observation [34, 37]. Ethnographic data included extensive field notes and reflexive journaling. Sharing circles and interviews were audio recorded, with translation from the local language to English in real‐time or during transcription by the cultural liaison. Transcription involved de‐identification of data, with assignment of participant numbers and data storage on a password‐protected and encrypted server.

Participants included mothers and key informants such as NGO advocates, community leaders, healthcare providers, and educators. With firsthand experience with albinism, they contributed rich descriptions of everyday life for mothers and children with albinism and detailed information about what contributed to mothers’ resilience, such as social inclusion and access to services. Because of travel disruption and the COVID‐19 pandemic, we conducted data collection in two phases. The first phase involved two field visits in Tanzania where we interviewed 61 participants (26 mothers and 35 key informants). In the second phase, we conducted virtual interviews (on digital platforms of Zoom or WhatsApp) with 36 participants (22 mothers and 14 key informants) in South Africa. The third phase (not included in this article) is beginning in Ghana since COVID‐19 pandemic restrictions have lifted. Of the mothers, 12 had albinism themselves and 36 were mothers of children with albinism. The final sample was composed of 97 participants, 21 of whom were PWA (see Table 1).

**TABLE 1:** Demographic characteristics.

Demographic characteristics	Tanzania (*n* = 61)	South Africa (*n* = 36)	Totals (*n* = 97)
Mothers^a^	(*n* = 26)	(*n* = 22)	(*n* = 48)
Mothers with albinism	9	3	12
Mothers with children with albinism	17	19	36
Rural	14	2	16
Urban	12	20	32
Employment status			
Collective income (i.e., mamas’ groups)	12	1	13
Subsistence income (e.g., selling fruit, milling grain, sewing, soap making)	1	1	2
Collective and subsistence income	8	—	8
Employment income	3	8	11
Homemaker	—	12	12
Retired	2	—	2
Religious affiliation			
Christian	19	21	40
Muslim	7	1	8
African traditional religion^b^	—	—	—
Other/None	—	—	—
Did not answer	—	—	—
Ancestry			
African descent	26	22	48
Non‐African descent	—	—	—
Key informants	**(*n* = 35)**	**(*n* = 14)**	**(*n* = 49)**
Occupation			
Healthcare providers (nurses, genetic counselors, dermatologist)	4	4	8
Social worker	1	1	2
NGOs	4	3	7
Faith leaders	18	1	19
Journalist	1	—	1
Policy makers	1	2	3
Teachers	5	—	5
Traditional healers	—	2	2
Researchers	1	1	2
Albinism status			
With albinism	4	5	9
Without albinism	31	9	40
Gender			
Female	18	8	26
Male	17	6	23
Religion			
Christian	31	8	39
Muslim	3	—	3
African traditional religion^b^	—	2	2
Other/None	1	1	2
Did not answer	—	3	3
Ancestry			
African descent	32	11	43
Non‐African descent	3	3	6

Abbreviation: NGO, non‐governmental organization.

^a^ Mothers are the primary participants in the study, with their standpoint foregrounded. All other participants are grouped under the term “key informants.” Many mothers were also active in overlapping roles, such as albinism advocates.

^b^ Although very few participants identified ATR as prime religion, there was evidence in participant interviews and observation of how ATR overlaps with mainstream religion.

We began qualitative data analysis by reading and re‐reading the data, followed by coding and thematic analysis, all accompanied by ethnographic writing. We used NVivo to generate a codebook and organized our data through line‐by‐line coding by two researchers. Codes were grouped into categories that were further analyzed to present a comprehensive thematic narrative. Team members were involved in data analysis to validate and extend preliminary findings through consultation and interpretation as they read representative transcripts and code summaries. This collaborative process strengthened interpretations of the interactions between mothers impacted by albinism and the structures that shaped their experiences. For this article, comparative analysis [38] with the two countries as case studies brought into relief promising practices that could be transferred and scaled.

### 2.1 Research Ethics and Scientific Quality

Research ethics clearance was obtained from the four Canadian universities of the co‐investigators; in Tanzania from National Institute of Medical Research (NIMR) and Tanzania Commission for Science and Technology (COSTECH); and in South Africa from University of Pretoria. Research materials such as consent forms were translated into local languages, and in cases where a participant was not able to read, the consent was read to them, and they provided verbal consent. Herr and Anderson's [39] five validity or quality criteria for action research also apply to critical ethnography (see Table 2).

**TABLE 2:** Quality criteria.

Description of quality criteria	Study‐specific examples
Dialogic validity comes in the form of peer review; that is, inquiry that is carried out collaboratively with other researchers has greater dialogic validity	Our research‐advocacy‐policy network provided a rich context of collaborative research. Frequent (online) meetings were held to discuss data collection and analysis
Catalytic validity refers to the dynamic nature of research, due to the transformation of understanding for both researcher and participants	Participants’ contributions brought about the researchers’ evolving understandings that guided an emergent design and interpretation. Participants expressed new insights that derived from their research involvement
Process validity is related to having a strong methodology that allows for triangulation of data and trustworthiness	The study had multiple data forms (transcripts, fieldnotes) from different settings and participants (mothers, key informants)
Democratic validity ensures that the outcomes of the research will benefit the community researched	Partnering with NGOs in each country facilitated contextually relevant research and integrated knowledge translation
Outcome validity refers to assessment of the achievement of community‐based goals	A public forum with 100 attendees was hosted in South Africa in 2023 with the release of the Project Report and the premiere of a short film validated the finding's resonance with the goals of local albinism organizations and governments

Abbreviation: NGO, non‐governmental organization.

## 3 Results

Tanzania and South Africa are individual case studies that provide us with insights into critical resilience about how these countries addressed the perilous challenges for persons impacted by albinism (see Table 3). These two countries are similar in population, but with different histories and political economic systems. Reflecting a higher Gross Domestic Product (GDP) and Human Development Index, infant mortality rates as an overall health indicator are lower in South Africa. Tanzania has been the epicenter of violence against PWAs, and the site where human rights atrocities were first reported. For mothers impacted by albinism in our study, these national differences translated into access to services, employment/income, and safety and security. In sum, in Tanzania mothers looked to government for safety; in South Africa mothers looked to government for services. Our analysis also shows remarkable congruence across the two countries, with mothers impacted by albinism facing formidable challenges as they carried the burden of the condition, whether as PWA themselves or as a parent to a child with albinism. Yet, most mothers in our study had found a way forward through these challenges—with a resilience that a Tanzanian female pastor described as *ujasiri* (Swahili), meaning to “Look danger in the eye and say, ‘I'll go through it’.” To better understand mothers’ transition to resilience and activism, we give voice to mothers as to their recommendations and distill promising practices (see Table 3). The distinct national and local contexts gave rise to variation in the relations between institutions and individuals, and among individuals that in turn contributed to critical resilience.

**TABLE 3:** Research sites and promising practices compared.

Demographics	Tanzania	South Africa
Population (as of 01/01/2023) [40]	65,657,000	62,797,000
Date of Independence	1961	1994
Colonizing Countries	Omani Arabs Portugal Germany Britain	Portugal the Netherlands Britain
Prevalence of Albinism [7]	1 in 1400 (hospital survey), 1 in 2673 (national census)	1 in 1100–3900 depending on the specific population
Human Development Index [41]	0.549	0.643
Gross Domestic Product [42]	$67.84 billion	$419 billion
Happy Planet Index [43]	28.8	29.3
Mortality rate, infant (per 1000 live births) [44]	34	26
**Mothers’ recommendations**	Mothers require positive birth education. Peer support groups equip mothers as human rights defenders. Community education is required about albinism, as a way to ensure safety. CSOs provide such education along with access to resources for eye and skin care screening (glasses and sunscreen). Faith leaders should be engaged as advocates. Mothers rely on community‐based support (informal) more so than government services that are inconsistent especially in rural areas	Mothers require accurate health information about albinism and call for support such as eye and skin care screening (glasses and sunscreen) more so than ensuring (physical) safety and security. Mothers have more recommendations for and expectations of the government “from the top” as duty bearers, especially for provision of disability grants
**Promising practices**	1. Peer support groups to build capacity as human rights defenders 2. International NGOs to fill gaps and advocate 3. Faith leaders as advocates	1. Legislative frameworks and government resources for human rights 2. Genetic counseling and health education to equip families to access resources 3. Traditional healers and Indigenous knowledge keepers as advocates

Abbreviation: NGO, non‐governmental organization.

## 4 Case I: Tanzania

In the early years of its independence from British rule, President Nyerere developed the governing philosophy referred to as *ujamaa*, meaning familyhood, with the goal of integrating rural villages into a modern state, such that all citizens have an institutional structure to allow them to participate in their local government [45]. Through this structure, organizing villages was to bring government facilities of health and education to their citizens and serve as groundwork for a unified nation that overcame ethnic and linguistic differences [46]. Despite the critiques of *ujumaa* as not delivering on its ideals, local governance was often referenced to in our fieldwork as a mechanism to ensure the safety of PWA, with mothers looking to street leaders for protection, though safety was not always provided, as evidenced by reported attacks against PWA. In contrast to South Africa, Tanzanian participants tended to not anchor their claims in legal frameworks, nor reference government as duty bearer. Rather, their reference points were predominantly fellow citizens and civil society. Three promising practices—peer support groups, international NGO support, and faith leaders’ advocacy—stood out as creating conditions for critical resilience.

### 4.1 Peer Support Groups Build Capacity as Human Rights Defenders

We were introduced to peer support groups for mothers impacted by albinism. *Upendo wa Mamas* (translated from Swahili to English as “the love of mothers”) and its organizers welcomed us to spend time with them in their Mwanza and Dar es Salaam locations [22]. Researchers spent days at their workshop where mothers made beeswax products for sale and collective profits. The mothers shared their experiences of living with albinism, whether themselves or their children. One mother observed that the group activities “earned them respect in society, because if you empower us, we are capable.” On a nearby island, mothers in another peer support group, also started by an international NGO, participated in making local arts, a singing group, and a sewing enterprise. During the year of our fieldwork, approximately 50 mothers were involved in these peer groups between three sites. In a sharing circle in rural Mwanza, a mother articulated the value of the peer support group and recommended it to others:

This kind of group is a very big help. It can feel like you are alone as a mother affected by albinism, but when you come together with others, you learn that you can manage. You understand that your child with albinism was born for a purpose. You get that education of understanding their value. You learn how to make a business. The togetherness reaches to a lot of areas.

Such sentiments were shared by many mothers, as well as key informants. Even as mothers might be abandoned by partners and shunned from their communities, they found ways to re‐create community on their own terms (see Ibhawoh et al. [47]) and become human rights defenders in their own right (see Ero et al. [48]).

### 4.2 International NGOs Fill Gaps and Advocate

Along with Tanzania Albinism Society, we conducted fieldwork with two international NGOs (Under the Same Sun, Standing Voice) that played a vital role in the safety and security of PWA. As violence against PWA escalated between 2009 and 2010, the Tanzanian government initially placed those most at risk in temporary holding centers [49]. Yet, placing these children in temporary holding centers still had them facing at a lack of security, care, and protection [49], and thus, the Tanzanian government needed to take more sustainable and effective steps to eliminate the violence against all children with albinism. As a result, with international attention to the plight of these children, international NGOs stepped in with child sponsorship at private boarding schools that ensured safety and provided quality education [50]. Hundreds of children received protection in this way until the attacks reduced, and they are now increasingly returning to their communities. A second gap that international NGOs stepped into involves skin and eye screening, with mobile clinics serving PWAs in rural contexts who could not access dermatology clinics in the city. Standing Voice, with its Skin Cancer Prevention Programme, has delivered sunscreen, skin screening, and early intervention in Tanzania and Malawi to nearly 9500 PWA, with a reduction in skin cancer rates by 23% [51].

Participants spoke about tensions in relation to the role of international NGOs. On the issue of child sponsorship, participants observed disrupted child–parent relationships and intra‐family relationships if not all siblings with albinism were sponsored. About health services, tensions had to do with health services not integrated into the national health system, and provision by foreign professionals without continuity or accountabilities. A community liaison and albinism advocate observed,

That's why they're called non‐governmental organizations. If we went to a (government) social worker and did not get the help then that would be a different case. But the way NGOs have done it, they've built so much, and we've built so many expectations from the organizations. Even they cannot handle these expectations.

Nonetheless, participants were unified in their assessment that, on balance, the contributions of international NGOs during dire times outweighed the tensions [49]. A mother reported, “I have no other place except Upenda wa Mamas. Anything to do with raising my child with albinism I have never had that opportunity until I reached out to this organization.” Mothers expressed relief and gratitude for sponsorship even as they experienced the grief of separation and loss of agency as a parent (see Strobell [22]). In the face of minimal government action (the UN Committee on the Rights of Persons with Disabilities (CRPD) has observed that Tanzania's lack of investigation and action is equivalent to condoning ritual killings and mutilations of people with albinism. See: https://www.ohchr.org/en/press‐releases/2024/04/tanzanias‐lack‐investigation‐and‐action‐equivalent‐condoning‐ritual‐killings. In September 2024, a landmark public hearing was presented to the African Court on Human and Peoples’ Rights against the United Republic of Tanzania (Application No. 019/2018), with a subsequent ruling that Tanzania failed to protect the human rights of persons with albinism. See: https://www.african-court.org/wpafc/african-court-concludes-public-hearing-in-application-no-019-2018-centre-for-human-rights-and-others-vs-tanzania-on-alleged-human-rights-violations-of-persons-with-albinism-in-tanzania/, The situation would have been more calamitous for hundreds of children had NGOs not intervened [49]. Sponsorship programs have saved lives and been instrumental in educating the next generation of PWA as leaders. In the gap of governments in low‐resource countries not providing fully for their citizens, international NGOs continue to have a role, albeit a contested role. In a globalized world and in the crisis of human rights atrocities, these organizations when working closely with local partners present as intermediary good practices.

### 4.3 Faith Leaders as Advocates

Beliefs about albinism in many African communities tend to be spiritualized, in a context where cosmology is rich in interactions between the natural and spiritual worlds [52]. Religious affiliation is high, for both the colonizing religions of Christianity and Islam, and for African Traditional Religion [33, 53]. Tanzanian participants repeatedly spoke about the influence of faith leaders, asking that they be more involved in the cause of albinism to leverage their influence. In a Tanzanian church, a faith leader had done just this, mobilizing the faith community to embrace a child and their family with albinism. Over time, contact with the child and their family brought about familiarity such that “everyone just loved the child.” Social inclusion spread into the community. Notably, in other situations, faith leaders unwittingly reinforced ostracizing attitudes towards PWA with their messages on witchcraft, and some were implicated in attacks against PWA. Therefore, faith leaders have in some cases been part of the problem, not the solution. A Tanzanian faith leader put it this way: “It is bad that the church did not respond when people with albinism were killed. The church remained silent, and did not condemn the act of killing people with albinism.” This leader went on to envision a better way “when the church works together with government and police, so we can serve mothers of children with albinism…the next generation will understand what is albinism, what is the right belief and what is not the right belief (about albinism).” According to the UN Independent Expert on Albinism, in a society where faith communities and faith leaders are important social actors, they are credible allies to leverage moral suasion against human rights violations in ways that other human rights defenders cannot (see Reimer‐Kirkham et al. [54]).

## 5 Case II: South Africa

Although smaller in population than Tanzania, South Africa enjoys a higher GDP and influence as a global player and a middle‐income country (see Table 3). As a result, mothers impacted by albinism had more access to resources, such as a disability grant and genetic counseling, both of which are minimally available in other sub‐Saharan countries. As in Tanzania, peer support facilitated by NGOs played a remarkable role in contributing to resilience for mothers impacted by albinism. Complementing and ensuring uptake of government resources were a multitude of NGOs and albinism advocates. A notable difference for South Africa is that these civil society organizations are for the most part local, regional, or national (i.e., not international). Peer support groups are innovative and entrepreneurial in leveraging decades of advocacy and the responsiveness of technology (e.g., a WhatsApp group for mothers affected by albinism). In contrast to Tanzania, South African mothers were more informed as to the genetic cause of the condition and less impacted by misbeliefs (see Reimer‐Kirkham et al. [21]; Strobell [22]), a finding that suggests the growing momentum and positive influence of South Africa's progressive health system and policy frameworks, genetic counseling, health professions education, and albinism advocacy (all as promising practices).

### 5.1 Progressive Health System and Policy Frameworks

Since the 1996 *South Africa Constitution*, government policies and legislative frameworks such as the *Promotion of Equality and Prevention of Unfair Discrimination Act* [55] have contributed to positive day‐to‐day effects for PWA. Especially when supported by a health navigator or albinism advocate, several mothers reported that they received the *South African Social Security Agency Care Dependency Grant* [56] for their child with albinism. A social worker explained:

Now the government is taking cognizance of the fact that albinism is a disability and trying to ensure that people with albinism are included in policies. The South African Constitution protects everyone. But remember these are unique challenges. There are no policies deliberately targeted to ensure that these mothers benefit from the social and economic pipeline. That is a huge gap that needs to be addressed.

Albinism advocates in South Africa took confidence in anchoring their work in the Constitution and guided mothers to apply for the *Care Dependency Grant* (South Africa Social Security Agency). There was variation as to whether the child would be deemed eligible, depending on government agents’ knowledge about albinism and whether they accepted the medical assessment of the child with albinism as having a severe disability requiring full‐time special care. This variation serves as example of the challenge of policy implementation and policy coherence to achieve the human rights of PWA.

South Africa's primary health system also builds into resilience for mothers impacted by albinism (see the White Paper for the Transformation of the Health System in South Africa [57] that outlines the policy objectives and principles for the Unified National Health System, including primary care). When compared to other lower resource countries in Africa such as Tanzania where access to healthcare was lacking or patchwork with NGO provision rather than government, South Africa's primary healthcare facilities meant that mothers had a good likelihood of a supported birth and government resources such as skin and eye care. Even in rural Northern Cape, a hospital had a dedicated dermatology albinism clinic once/month. A small local NGO run by a mother, herself impacted by albinism, ensured that new parents could access these services. She was known as the local advocate and lay counselor and would be phoned by the hospital if a child with albinism was born.

South Africa has high rates of gender inequality and violence, a national pattern that impacted the mothers in our study. In 2019, the national cabinet adopted a framework that obligates all spheres of government to undertake and report on gender‐responsive program planning, budgeting, and implementation and utilize disaggregated data to inform their strategic planning and annual work plans (see *Gender Responsive Planning, Budgeting, Monitoring, Evaluation*, *and Auditing Framework* [58] and *Strategy for Economic Empowerment of Women Youth and Persons with Disabilities* [59]). Oversight structures monitor implementation and hold government and state entities to account to ensure compliance. A strategy that requires that all government departments budget, plan, and report for gender, youth, and disabilities was adopted (a *National Strategy on Gender‐based Violence and Femicide*). As a promising practice, government intervention with funding and accountabilities to ensure service delivery; economic inclusion of women, youth, and persons with disabilities; eradication of gender‐based violence and femicide; and social transformation can go far to pave a pathway to resilience for mothers impacted by albinism, particularly when civil society and government services facilitate access to funds and programs.

### 5.2 Genetic Counseling and Health Education Equip Families

Contrasting to Tanzania where mothers described traumatic experiences of discrimination and abandonment at the time of giving birth to a child with albinism, South Africa fieldwork provided exemplars (though not the majority) of positive birth experiences. Participants who accessed a genetics clinic in Johannesburg had strikingly better experiences (see Reimer‐Kirkham et al. [21]; Kromberg and Kerr [60]). Consider the case of one mother: Although she was surprised by albinism, she was immediately told by the birth attendant about its genetic origin and that the baby would be able to thrive. The need for eye and skin care was explained, and she received a referral to the genetics clinic within the first days and was seen regularly thereafter. Her husband attended with her, and together they joyfully embraced parenting. Another mother observed she used to receive sunscreen from a genetics clinic, though she subsequently had to pay out of pocket and rely on her mother and in‐laws to help her find sunscreen. South Africa is the only country in Africa where genetic counseling is offered (albeit mostly accessible in urban areas), suggesting the need for a primary level of genetic counseling that could be provided by all healthcare workers encountering PWA and more accessible online resources, given that mothers described “googling” for albinism information [21].

### 5.3 Traditional Healers and Indigenous Knowledge Holders as Advocates

In South Africa, the albinism movement, including a country visit by the UN Independent Expert on Albinism, has engaged traditional healers as they are seen as potential allies in shifting community misperceptions against PWA. This positive engagement contrasts to Tanzania where traditional healers were implicated with harmful practices related to accusations of witchcraft and ritual attacks against PWA (see United Nations General Assembly [61]; Reimer‐Kirkham et al. [62]). Like faith leaders in Tanzania who became human rights defenders, our interviews with traditional healers, along with Ero's investigations as UN Independent Expert [61], suggest that they can be a voice for good, rather than harm. Consider the experience of one mother:

There's a lady who I'm seeing, she's a traditional healer. I went there before I gave birth, and she told me that I'm carrying a blessing from my ancestors. And then I gave birth only to find out that my child has albinism. She told me that “yeah, I told you that your kid will be a blessing and now you see, this is a blessing.”

Although this mother received the traditional healer's comment positively, an underlying message may be that a “blessing” infers a mystical component that might make PWA vulnerable to attacks for their body parts believed to have special spiritual powers (see Ibhawoh et al. [47]). The two traditional healers we interviewed were aware of acts of violence against PWA but asserted that PWA are human beings, with one differentiating those who perform “barbaric acts” of violence against PWA as not practicing traditional medicine. This differentiation might be due to the promulgated *Traditional Health Practitioners Act 22* of 2007 [63], which provides a framework for regulating traditional healers and Indigenous knowledge holders’ practices in South Africa.

Engaging traditional healers and Indigenous knowledge holders carried considerable complexity. Many participants voiced fear and condemnation of witchcraft and its association with traditional healers—specifically their perpetuation of untruths about albinism and their perceived involvement in violence against PWA. In Tanzania, we heard firsthand accounts of attacks against PWA. Despite age‐old fears and propaganda instigated by the *South African Witchcraft Suppression Amendment Act 50* of 1970 [64] on local Indigenous practices, there were fewer reports of attacks in South Africa. Nonetheless, mothers impacted by albinism expressed ever‐present fear of mistreatment and violence. Along with the concern for harmful practices, some participants worried that PWAs’ skin cancer is mistreated with herbal remedies, and that allopathic medicine is only sought out when the cancer is advanced. Given the influence of traditional healers, calls for decolonizing approaches to Indigenous knowledges and other forms of knowledge need to be explored in the South African context [65]. With the urgency of addressing all sources of human rights violations against PWA, engagement with regulated traditional healers can be a promising practice.

### 5.4 Limitations

These findings should be considered alongside the study's limitations, which relate largely to adjustments required during the pandemic. Because of limited travel, we were unable to conduct in‐person fieldwork in South Africa, which would have facilitated a larger and more representative sample. This limitation was offset by the intersectional nature of our team, with strong local partnering that contextualized data analysis processes [66, 67].

## 6 Conclusion

Data from this international, policy‐relevant ethnographic [68] project has allowed us to take a broad view of the resilience of mothers impacted by albinism. Despite difficult circumstances and intersecting structural constraints, many mothers affected by albinism came to display resilience. Fundamentally, their resilience was not a matter of them “bouncing back” following a life disruption or adversity. Mothers were already structurally vulnerable; albinism exacerbated their deprivation and social exclusion. And yet—as they received the structural resources of peer support and economic empowerment, access to health information and healthcare, social inclusion, and government responsiveness—they came to thrive. Albinism itself was manageable in the right circumstances. We interpret this as *critical* resilience [29], in that individual experiences are understood in relation to social relations of power (advantage and disadvantage) and accompanying social structures (such as poverty and gender inequality) are recognized for their detrimental consequences, and are resisted and ameliorated. Health and gender equity are intertwined [69] for mothers impacted by albinism who had incisive recommendations as to the conditions they required for resilience (see Table 3). Drawing on African feminist Tamale [33], resilience is not primarily a possession of mothers, but rather a social resilience comprised by the structure and quality of the social relationships, collective practices, and identities present in communities (local and national) [70]. Amo‐Agyemang [70] calls for decolonizing discourses of resilience to account for diverse African communities. Our analysis has shown variations between and within Tanzania and South Africa in the structural factors and promising practices that create conditions for resilience for mothers impacted by albinism.

Our findings also reveal the concurrent operations of universal and local human rights as lived in the everyday. Mothers in our study were active as human rights defenders (see Ero et al. [48]), acting on an intuitive sense of justice where they spoke up on behalf of themselves and their children. Even as they were expelled from their communities by husbands and extended family, peer support groups became sites of similar loyalties and means of livelihood (see Ibhawoh et al. [47]). Grounding human rights norms in local communities (i.e., vernacularizing human rights) requires that subaltern actors such as the mothers in our study be seen as human rights claimants and defenders. Their grassroots actions, along with advocates such as faith leaders and traditional healers, create environments in which state actors are more likely to be held accountable for the implementation of universal human rights. Our work illustrates how a dual stream is required, of bottom‐up advocacy and local response to human rights violations, along with top‐down human rights initiatives such as strengthened legislative frameworks (e.g., the recent *African Union continent‐wide Plan of Action on Albinism* [71]). International conventions exist to protect the rights of PWA, including the *Convention on the Rights of Persons with Disabilities* [72], which enabled a shift from charity, welfare, and medical approaches to disability to a social and rights‐based approach. The violence, discrimination, and other human rights violations faced by mothers impacted by albinism are not primarily because of a lack of suitable legal and policy frameworks but rather due to the practice of human rights and the implementation (or lack thereof) of the sociocultural frameworks of their everyday lives [73]. The promising practices distilled from the Tanzanian and South African cases should be considered for broader uptake, importantly with local adaptations. Concrete, prioritized initiatives based upon research‐derived promising practices and implemented through intersectoral collaboration can go far in creating the conditions for thriving families and communities impacted by albinism.

## Author Contributions

Funding acquisition: Sheryl Reimer‐Kirkham, Ikponwosa Ero, and Barbara Astle. Conceptualization, methodology, and formal analysis: Sheryl Reimer‐Kirkham, Ikponwosa Ero, and Barbara Astle. Original draft preparation of manuscript: Sheryl Reimer‐Kirkham. Data curation: Sheryl Reimer‐Kirkham, Barbara Astle, Emma Strobell, and Meghann Buyco. Coding: Emma Strobell and Meghann Buyco. Formal analysis, interpretation of results, and writing – review and editing: Sheryl Reimer‐Kirkham, Barbara Astle, Ikponwosa Ero, Lori Beaman, Maretha de Waal, Bonny Ibhawoh, Elvis Imafidon, Jennifer Kromberg, Ronell Leech, Nomasonto Mazibuko, Innocentia Mgijima‐Konopi, Ramadimetja Shirley Mooa, Dianah Msipa, Perpetua Senkoro, Sonya Sharma, Wisdom Tettey, Mpho Tjope, Boateng Wiafe, Meghann Buyco, and Emma Strobell. All authors reviewed the results and approved the final version of the manuscript.

## Funding

This study was funded by the Social Sciences and Humanities Council of Canada (#435‐2019‐1120, 2019–2023).

## Ethics Statement

The research adheres to the ethical policies of the journal. Research ethics clearance was obtained from four universities in Canada (Trinity Western University, University of Toronto, University of Ottawa, and McMaster University); in Tanzania from National Institute of Medical Research (NIMR) and Tanzania Commission for Science and Technology (COSTECH); and in South Africa from University of Pretoria.

## Consent

Informed consent was provided by all participants included in this study, in accordance with ethics approvals.

## Conflicts of Interest

The authors declare no conflicts of interest.

## Data Availability

The data generated and analyzed during the current study are available from the corresponding author upon reasonable request.
